# A research priority setting for ankle-foot orthosis care in the Netherlands; a survey among rehabilitation specialists, orthotists, and orthotic users

**DOI:** 10.1097/PXR.0000000000000499

**Published:** 2025-10-29

**Authors:** Niels F. J. Waterval, Fieke S. Koopman, Frans Nollet, Merel A. Brehm

**Affiliations:** 1 Department of Rehabilitation Medicine, Amsterdam UMC Location University of Amsterdam, Amsterdam, The Netherlands; 2 Amsterdam Movement Sciences, Rehabilitation and Development, Amsterdam, The Netherlands

**Keywords:** ankle foot orthoses, rehabilitation, research priority, research agenda, orthosis

## Abstract

**Background and Objective:**

Ankle-foot orthoses (AFOs) are often prescribed for lower leg muscle impairments. In order to conduct impactful research, we aimed to establish a list of prioritized research questions regarding AFO care in the Netherlands from the perspective of healthcare professionals and AFO users.

**Study Design:**

A purposefully designed online survey.

**Methods:**

To identify relevant research questions regarding the AFO care process the online survey was sent to rehabilitation specialists, orthotists and AFO users in the Netherlands. The research questions were grouped into specific research needs according to the Process description of medical devices. The priority of the research needs was determined using a 11-point numeric rating scale (NRS), with 10 being the highest priority.

**Results:**

The survey was completed by 62 rehabilitation specialists, 47 orthotists and 184 AFO users. Rehabilitation specialists prioritized research on “Effects of AFO properties” (numeric rating scale score: 8.3 ± 1.2) and “Choice of AFO type and design” (8.1 ± 2.0). Orthotists prioritized research on “How to cast and correct the plaster model” (8.9 ± 1.3) and “Effects of AFO properties” (8.7±1.4). Ankle-foot orthoses users prioritized research on “Improvement of AFO-shoe fitting and information on appropriate shoes'” (8.6 ± 1.8) and “Possibilities to choose and test different AFOs before provision” (8.5 ± 1.3).

**Conclusions:**

Our inventory of research needs has provided a list of research priorities that can be used to structure AFO-related knowledge development. Healthcare professionals and patients have different research priorities that should be taken into account when designing and setting up future AFO-related research.

## Introduction

Neuromuscular diseases, brain damage (including stroke, cerebral palsy, and MS), and trauma can all result in lower leg muscle impairments, such as weakness and/or spasticity. These impairments often limit walking ability and lead to difficulties in performing activities of daily living.^1-4^ Ankle-foot orthoses (AFOs) can be used to improve walking ability. Ankle-foot orthoses are assistive medical devices that encompass (part of) the foot and ankle joint and restrict ankle movement by generating an external passive torque, which, in turn, supports the lower leg muscles^5^ and can improve walking ability.

**Figure 1.**
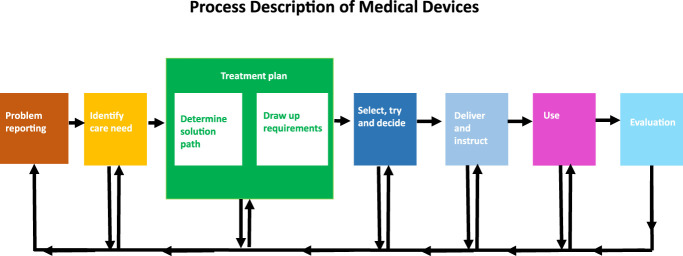
Schematic overview of process description of medical devices.

The provision of AFOs in Dutch rehabilitation care is multidisciplinary in nature and usually involves at least a rehabilitation specialist, an orthotist and the (potential) AFO user. According to the Process Description of Medical Devices,^6^ the orthotic care process consists of several steps (Figure 1). These steps include problem reporting; identification of the care need; creating the care plan; selecting, trying, and deciding on the orthosis; delivery and instruction of the orthosis; using the orthosis; and the evaluation. Standardized protocols for each step of the care process have been developed and described in the Dutch guideline for the provision of lower limb orthoses in people with neuromuscular diseases to support clinical decision making.^7^

Ankle-foot orthoses provided in Dutch rehabilitation practice have been shown to significantly improve walking ability and physical functioning in people with neuromuscular disorders.^8,9^ However, despite their effectiveness, a high percentage of AFO users reported to be dissatisfied or to discontinue using their AFO.^10,11^ This may be partly explained by the lack of scientific evidence underlying the different steps of the AFO-care process, leading to variability in AFO care and suboptimal AFO prescriptions.^12^

To improve the quality of AFO care in the Netherlands, more scientific knowledge about the different steps in the care process is needed. To initiate meaningful research and ensure its relevance to clinical practice, the involvement of relevant stakeholders, such as rehabilitation specialists, orthotists, and AFO users, is required. A first step toward such engagement is to assess and prioritize their research needs. In this study, using a survey-based approach, we aimed to identify and cluster research questions into research needs and establish a comprehensive list of prioritized clinical questions regarding AFO care in the Netherlands from the perspective of health care professionals and AFO users.

## Methods

A cross-sectional study was conducted using 2 online surveys. First, we inventoried research questions among rehabilitation specialists, orthotists, and AFO users as part of an online survey about the organization of AFO care in the Netherlands. After clustering the research questions into research needs, according to the different steps as distinguished in the Process Description of Medical Devices, a second online survey was sent out to prioritize the identified research needs. The reporting of our study followed the Checklist for Reporting of Survey Studies.^13^ The research was exempt from formal medical ethical approval under the Dutch law.

### Creation of the survey

As there were no validated questionnaires that addressed our study objectives, we deliberately developed a web-based survey using Castor EDC (Amsterdam, The Netherlands) to identify AFO-related research needs.^14^ Separate surveys were created for rehabilitation specialists, orthotists, and AFO users, and the survey took approximately 15 minutes to complete. The survey for rehabilitation specialists and orthotists consisted of 7 questions about work environment, specialty, and years of experience, 30 questions about the orthotic care process (outside the scope of this study), and open-ended questions about whether they had AFO-related research questions for each specific step of the orthotic care process. The survey for AFO users consisted of 8 questions about their age, living situation, diagnosis, duration of AFO use (years), and the prescribed type of AFO (custom or prefabricated), followed by 51 questions about the orthotic care process and the perceived effectiveness (outside the scope of this study), and 5 open-ended questions about whether they had any research questions related to the different steps of the care process. All 3 surveys were in Dutch. The translated questions can be found in Appendix 1, Supplemental Digital Content 1, http://links.lww.com/POI/A346. No restriction on whether Dutch was the mother language was applied. All respondents could indicate whether they would be willing to participate in a second survey to identify research priorities. Before distribution, each survey was pretested by 2 rehabilitation specialists, 2 orthotists, and 2 AFO users. Their feedback on textual ambiguities and potential missing topics was incorporated into the final version of the surveys.

Respondents who agreed to participate in the research priority setting were sent a second online survey via Castor EDC. In this survey, respondents were asked to prioritize the identified research needs on a 10-point numerical rating scale (NRS, where 10 points means it has the highest priority), and also to select the 3 main topics they considered most important.

### Distribution of the survey

The first survey to inventory research questions was sent to rehabilitation specialists, orthotists, and AFO users. Regarding rehabilitation specialists, the survey was distributed via the Netherlands Society for Rehabilitation Medicine (Vereniging voor Revalidatie Artsen [Dutch]). An email with the invitation to participate and a link to the survey was sent to the 318 members involved in rehabilitation care of neuromuscular diseases, brain damage (including stroke, cerebral palsy, and MS), and trauma. Although AFOs are frequently provided in these patient groups, not all rehabilitation specialists are routinely involved in AFO care. In the invitation email, it was, therefore, explicitly stated that the survey was intended for specialists involved in Dutch AFO care.

The survey for orthotists was distributed by email to the members of the Dutch Professional Association of Orthopaedic Technology (Nederlandse Beroepsvereniging Orthopedisch Technologen [in Dutch]), which includes approximately 60 orthotists. In addition, an invitation to distribute the survey among orthopedic technicians was send to companies associated with the Foundation of Orthopedic Aids in the Netherlands (Stichting Orthopedische Hulpmiddelen Nederland [in Dutch]). This foundation consists of more than 50 partner companies, most of which have multiple locations throughout the Netherlands. In the invitation, it was specified that the participating orthotist had to provide AFOs on a regular basis and that multiple orthotists could participate from one company.

The survey for the patients was distributed through the MS-patient association (MS-vereniging [in Dutch]) and the Dutch patients association for Neuromuscular Diseases (Spierziekten Nederland [in Dutch]) by email. In the invitation, it was explicitly stated that the survey was intended for current or previous AFO users. To control for multiple participation, which could potentially occur because the survey was distributed via different channels and the survey link could be opened multiple times, it was checked whether there were duplicates of completed questionnaires. All surveys were open for 1 month (between December 2022 and February 2023). No reminder was sent.

The second survey to prioritize the research needs was sent by email to all participants who agreed to be contacted. The survey was open for 1 month (from May to June 2023) and a reminder was sent after 1 week.

### Analyses

Data were exported from Castor EDC to SPSS (SPSS Inc, Chicago, United States) (v.28) for cleaning and analysis. Demographic characteristics of the respondents were described by calculating frequencies and percentages for categorical responses or means and standard deviations for continuous outcomes.

To formulate the research needs, the collected questions were clustered by one reviewer (N.W.). All questions were categorized according to the step of the Process Description of Medical Devices^6^ to which the question related. The steps are problem reporting; identification of the care need; creating the care plan; selecting, trying, and deciding on the orthosis; delivery and instruction of the orthosis; using the orthosis; and the evaluation. Subsequently, 2 reviewers (N.W. and F.K.) inductively clustered the research questions per step of the process description. Disagreements in clustering were discussed and resolved with a third reviewer (M.B.) if necessary. Each cluster was considered to be a research need. The naming of the research need was based on terms that best captured the underlying questions and was based on consensus among the research team.

To prioritize the research needs, means and standard deviations of the NRS scores per research need were calculated. As the scores are given on a 11-point scale, data were treated as numerical. Research needs were ranked on the basis of mean NRS scores. In addition, the percentage of respondents who selected the research need in their top 3 was determined. No statistical test was used to assess differences between needs.

## Results

### Respondent characteristics

The first survey was completed by 62 rehabilitation specialists (response rate 19.2%), 47 orthotists, and 184 AFO users. The response rate for orthotists and AFO users could not be determined because the number of orthotists invited through Stichting Orthopedische Hulpmiddelen Nederland and the orthopedic companies could not be established, and the number of AFO users among the members of MS-vereniging and Spierziekten Nederland was unknown. Most rehabilitation specialists (77%) and orthotists (70%) had more than 5 years of experience in AFO care and treated multiple diagnosis (Table 1). Of the 184 AFO users (105 female/74 male/5 missing), 79 were diagnosed with MS and 105 with a neuromuscular disease. Ankle-foot orthoses were used unilaterally by 80 participants and bilaterally by 75 participants. Twenty-nine participants (15.7%) had stopped using their AFO. Participants used AFO(s) for a mean of 7.2 ± 9.2 years, and the majority currently used a custom-made AFO (67.2%).

**Table 1.**
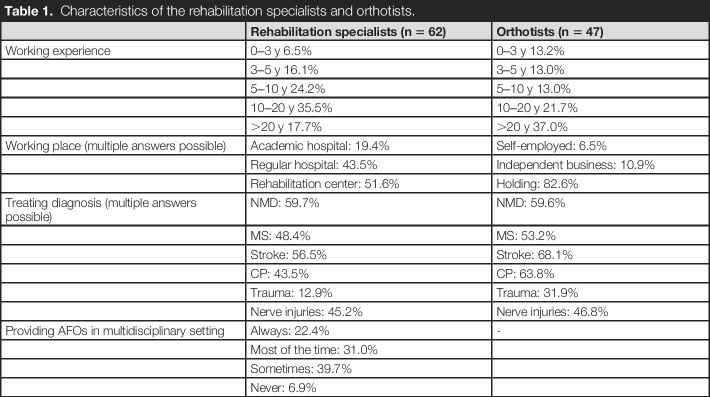
Characteristics of the rehabilitation specialists and orthotists.

	Rehabilitation specialists (n = 62)	Orthotists (n = 47)
Working experience	0‒3 y 6.5%	0‒3 y 13.2%
	3‒5 y 16.1%	3‒5 y 13.0%
	5‒10 y 24.2%	5‒10 y 13.0%
	10‒20 y 35.5%	10‒20 y 21.7%
	>20 y 17.7%	>20 y 37.0%
Working place (multiple answers possible)	Academic hospital: 19.4%	Self-employed: 6.5%
	Regular hospital: 43.5%	Independent business: 10.9%
	Rehabilitation center: 51.6%	Holding: 82.6%
Treating diagnosis (multiple answers possible)	NMD: 59.7%	NMD: 59.6%
	MS: 48.4%	MS: 53.2%
	Stroke: 56.5%	Stroke: 68.1%
	CP: 43.5%	CP: 63.8%
	Trauma: 12.9%	Trauma: 31.9%
	Nerve injuries: 45.2%	Nerve injuries: 46.8%
Providing AFOs in multidisciplinary setting	Always: 22.4%	-
	Most of the time: 31.0%	
	Sometimes: 39.7%	
	Never: 6.9%	

The response rate of the second survey was 75% (15/20) for the rehabilitation specialists, 58% (7/12) for the orthotists, and 79% (66/84) for the AFO users.

### Research needs

Rehabilitation specialists raised 48 research questions. After clustering, 8 distinct research needs were identified for 3 steps in the orthotic care process: creation of care plan, use of the AFO, and evaluation. Orthotists reported 28 research questions, from which also 8 research needs were identified for 5 steps in the orthotic care process, with the exception of problem reporting and identification of the care need. Ankle-foot orthoses users reported 101 research questions, covering 9 research needs for 5 steps of the orthotic care process, excluding problem reporting and identification of the care need. All questions are listed in Appendix 2, Supplemental Digital Content 1, http://links.lww.com/POI/A347. The research needs identified among health care professionals and patients, tabulated according to the Process Description of Medical Devices, are listed in Table 2. Four identical research needs were identified among rehabilitation specialists and orthotists; “Which AFO is most suitable for the individual,” “Effects of AFO properties on gait,” “AFO adherence,” and “Efficiency of custom-made AFOs.” There was no overlap in research needs between the health care professionals and AFO users.

**Table 2.**
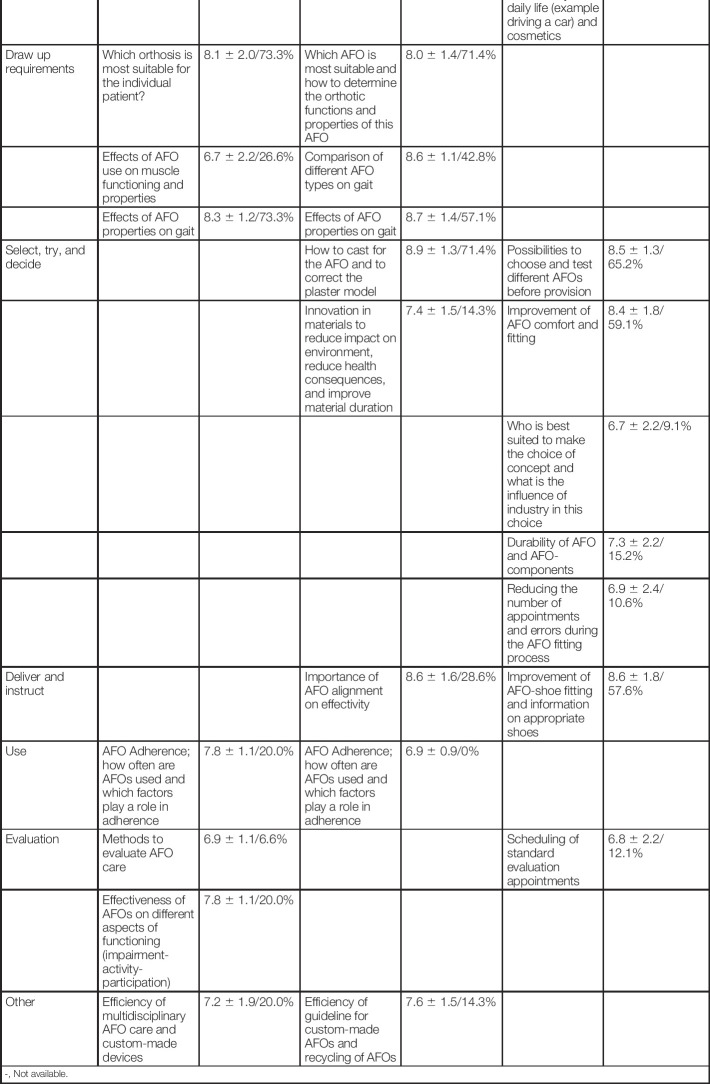
Overview of research needs by each group.

Step in prescription process medical devices	Rehabilitation specialists		Orthotists		AFO users	
	Research need	Importance score (NRS)/% rehabilitation specialist that placed research need in top 3 most important	Research need	Importance score (NRS)/% orthotist that placed research need in top 3 most important	Research need	Importance score (NRS)/% AFO users that placed research need in top 3 most important
Problem reporting	-		-		-	
Identify care need	-		-		-	
Determine solution path	When is an AFO indicated and which measurements are needed for indication setting?	7.5 ± 1.2/60.0%			Provision of information during the AFO care process	7.4 ± 1.8/16.7%
					Improving AFO functionality in daily life (example driving a car) and cosmetics	7.8 ± 2.0/57.6%
Draw up requirements	Which orthosis is most suitable for the individual patient?	8.1 ± 2.0/73.3%	Which AFO is most suitable and how to determine the orthotic functions and properties of this AFO	8.0 ± 1.4/71.4%		
	Effects of AFO use on muscle functioning and properties	6.7 ± 2.2/26.6%	Comparison of different AFO types on gait	8.6 ± 1.1/42.8%		
	Effects of AFO properties on gait	8.3 ± 1.2/73.3%	Effects of AFO properties on gait	8.7 ± 1.4/57.1%		
Select, try, and decide			How to cast for the AFO and to correct the plaster model	8.9 ± 1.3/71.4%	Possibilities to choose and test different AFOs before provision	8.5 ± 1.3/65.2%
			Innovation in materials to reduce impact on environment, reduce health consequences, and improve material duration	7.4 ± 1.5/14.3%	Improvement of AFO comfort and fitting	8.4 ± 1.8/59.1%
					Who is best suited to make the choice of concept and what is the influence of industry in this choice	6.7 ± 2.2/9.1%
					Durability of AFO and AFO-components	7.3 ± 2.2/15.2%
					Reducing the number of appointments and errors during the AFO fitting process	6.9 ± 2.4/10.6%
Deliver and instruct			Importance of AFO alignment on effectivity	8.6 ± 1.6/28.6%	Improvement of AFO-shoe fitting and information on appropriate shoes	8.6 ± 1.8/57.6%
Use	AFO Adherence; how often are AFOs used and which factors play a role in adherence	7.8 ± 1.1/20.0%	AFO Adherence; how often are AFOs used and which factors play a role in adherence	6.9 ± 0.9/0%		
Evaluation	Methods to evaluate AFO care	6.9 ± 1.1/6.6%			Scheduling of standard evaluation appointments	6.8 ± 2.2/12.1%
	Effectiveness of AFOs on different aspects of functioning (impairment-activity-participation)	7.8 ± 1.1/20.0%				
Other	Efficiency of multidisciplinary AFO care and custom-made devices	7.2 ± 1.9/20.0%	Efficiency of guideline for custom-made AFOs and recycling of AFOs	7.6 ± 1.5/14.3%		

-, Not available.

### Priority setting

For rehabilitation specialists, the research needs with the highest NRS score were “Effects of AFO properties on gait” (NRS score: 8.3 ± 1.2), “Choice of AFO type and design” (8.1 ± 2.0), and “AFO adherence” (7.8 ± 1.1). The top 3 most frequently selected important research needs were “Effects of AFO properties on gait” (11/15), “Choice of AFO type and design” (11/15), and “when is an AFO indicated” (9/15).

In the orthotist group, “How to cast and correct the plaster model” (8.9 ± 1.3), “Effects of AFO properties on gait” (8.7 ± 1.4), and “Comparison of different AFO types” (8.6 ± 1.3) were rated as most important, and “How to cast and correct the plaster model” (5/7), “Choice of AFO type and design” (5/7), and “Effects of AFO properties on gait” (4/7) were most frequently selected in the top 3.

**Figure 2.**
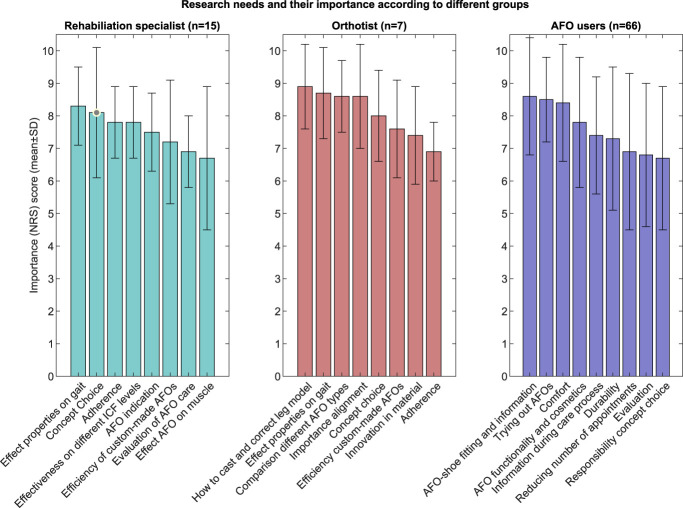
Numerical rating scores of the different research needs.

AFO users rated “Improvement of AFO-shoe fitting and information on appropriate shoes” (8.6 ± 1.8), “Possibilities to choose and test different AFOs before provision” (8.5 ± 1.3), and “Improvement of AFO comfort and fitting” (8.4 ± 1.8) as most important. These were also the most frequently selected research needs in the top 3 (38‒43/66) (Table 2 and Figure 2).

## Discussion

In this cross-sectional, survey-based study, we inventoried and prioritized the research needs related to AFO care in the Netherlands from the perspective of both health care professionals and orthotic users. A total of 177 research questions were posed, and these were grouped into 21 unique research needs across rehabilitation physicians, orthotists, and AFO users. Health care professionals, consisting of rehabilitation specialists and orthotists, prioritized research needs on how to select the AFO for the individual and improve its effectiveness, whereas users prioritized research needs on AFO fitting, trying-out, and comfort.

The research needs of rehabilitation specialists and orthotist were classified into different steps of the care process, where rehabilitation specialists mainly had needs in the steps “creation of the care plan,” “use,” and “evaluation,” whereas for the orthotists reported research needs in the steps “selecting, trying and deciding,” and “deliver and instruct.” These differences are in line with the different roles and tasks of the rehabilitation specialists and orthotists within the care process, as outlined in the Dutch guideline.^7^ Combined, these needs cover all 7 steps of the orthotic care process, except the first 2 steps, problem reporting and identification of the care need, indicating the scarcity of scientific evidence underlying current prescription guidelines.^7^ Despite the different roles, both groups identified the effect of AFO properties on gait, efficiency of custom-made AFOs, and AFO adherence as research needs, possibly because both groups are aware of the ineffectiveness of prescribed AFOs in clinical practice,^12,15^ and the relatively high nonuse of AFOs.^11,16,17^ The research needs also align with quality of care topics found extremely important by the large majority of orthotists (>80%) from a survey conducted in the United States, such as device usage, device characteristics, and patient communication.^18^ The prioritization of research on AFO properties could be related to the fact that most studies experimentally evaluating AFOs oftentimes lack detailed descriptions of their properties and materials,^19^ while it is known that such properties have a large effect on biomechanical outcomes.^20^ The research needs of the AFO users differed significantly from those of rehabilitation specialists and orthotists. Users prioritized research on AFO selection, the AFO-shoe combination, and AFO comfort. These research needs may not be surprising, as these topics are a major source of dissatisfaction among AFO users, as previously reported in 2 surveys of a diverse population of AFO users^21^ and people with Charcot-Marie-Tooth disease.^10^ In these surveys, up to 50% of AFO users reported discomfort, pain, and/or fitting problems. In addition, dissatisfaction with comfort and fitting, together with a lack of effectiveness, are important reasons for AFO discontinuation in various patient groups.^10,11,22^ Given these findings, it is surprising that comfort was not identified as a research need by rehabilitation specialists and orthotists, while improving the effectiveness and the properties of the AFO were given the highest priority. Furthermore, aesthetics and social acceptance of AFO design have been found to be important factors for adherence in children with cerebral palsy.^23,24^ The fact that systematic evaluation is not always part of the care process may reduce awareness of the prevalence of comfort problems.^25^ According to our data, conducting research into improving the fitting of AFOs within the shoes, personalization of the AFO design, and padding materials/location is needed to improve orthotic treatment outcomes.

The fact that users identified different needs compared to health professionals highlights the importance of involving AFO users in the development of AFO-related research. That professionals and patients have different interests is consistent with studies showing that research topics do not match patients' interest, as demonstrated for example in knee osteoarthritis.^26^ Users have experience-based knowledge, which may differ from the experience of health care professionals. Based on our research, it appears that health care providers are primarily focused on optimizing functioning with the AFO, whereas users are more focused on the ease of use and social acceptance. This difference is similar to the quality of care perspectives of both clinicians and users in the United States,^27^ and early inclusion of the patient perspective in AFO related research is, therefore, needed to improve the feasibility and implementation of research outcomes.^28^ In the context of AFO research, greater emphasis could be placed on patient-reported outcomes, which have been shown to have acceptable sensitivity and validity.^29,30^

This is the first research priority setting in AFO care that has the potential to structure and shape scientific advances AFO-related research. Although specifically conducted in the Netherlands, the results are likely to be applicable to other high-income care settings. We included the perspective of both health care professionals and users, resulting in a comprehensive overview of the research needs. We hope that in the future, our research priorities will be used by professional and patient societies, such as the International Society of Prosthetics and Orthotics, together with funding agencies to support the development of research agendas and projects. In the meantime, it seems important to continue to investigate the role of AFO properties^31^ and the AFO-footwear combination^32^ on not only on efficacy but also on patient-experienced outcomes such as comfort and usability.

Our study was limited to Dutch orthotic and health care system. In other countries, treatment protocols and provision of AFOs may differ.^33^ Generalizing the results internationally is further complicated by the lack of standardized terminology or use of outcome measures in AFO care. Standardizing terminology^34^ and outcome measures^35^ may also help collaboration between health professionals and users in AFO-related research and seems, therefore, important. In addition, our study may be susceptible to selection bias because of the limited number of responses and self-selection of participants. Participants were invited by a general invitation in Dutch, and the number of invited rehabilitation specialists, orthotists, and AFO users who met the inclusion criteria is unknown, making it impossible to calculate the response rate. Nevertheless, the number of responses seems acceptable given the total workforce in the Netherlands for the first questionnaire and the fact that the responding rehabilitation specialists and orthotists were diverse in experience and work location. Also the AFO users were diverse in disease and use of the AFO, but as our survey was only available in Dutch, people with insufficient knowledge of Dutch and likely different cultural background may be limited. The priority setting had less participants, especially in the orthotists group, and may be more susceptible to self-selection. It is possible that only people with a strong interest and commitment to improving AFO care responded, which may have led to specific research needs and other prioritization. For example, these committed participants may have been be more aware of the recent scientific literature regarding the impact of properties or inadequate indication settings, thus increasing their prioritization. However, as the most committed participants are possibly also the frequent providers, the results may still reflect the needs of the workforce.

In conclusion, our inventory of research needs has provided a list of research priorities that can be used to structure AFO-related knowledge development. Health care professionals and patients have different research priorities, highlighting the need to listen to all stakeholders when designing AFO research to ensure clinical applicability and impact.

## Funding

The authors disclosed receipt of the following financial support for the research, authorship, and/or publication of this article: This work was supported by ZonMW (grant number: 10310032110004).

## Declaration of conflicting interest

The authors disclosed no potential conflicts of interest with respect to the research, authorship, and/ or publication of this article.

## Supplemental material

Supplemental material for this article is available in this article. Direct URL citation appears in the text and is provided in the HTML and PDF versions of this article on the journal’s Web site (www.POIjournal.org).

## Supplementary Material
