# Supplementary material for: A research priority setting for ankle-foot orthosis care in the Netherlands; a survey among rehabilitation specialists, orthotists, and orthotic users
Source: Prosthet Orthot Int. 2025 Oct 29;50(4):410–7. doi: 10.1097/PXR.0000000000000499 (PMC13440560; doi:10.1097/PXR.0000000000000499)
Supplement: Supplementary file 1 [file poi-50-410-s001.docx]

**Appendix 1. Translation of the analyzed survey questions:**

**Rehabilitation specialists / Orthotist**

1. Year of birth
   a. open field
2. Gender
   a. option: Male / Female
3. In which setting do you work? (multiple answers possible)
   a. Options: General Hosiptal / Academic hospital/ Rehabilitation center
4. In which province do you work?
   a. Options: Noord-Holland/ Zuid-Holland/ Utrecht/ Brabant/ Zeeland/ Limburg/ Gelderland/ Overijssel/ Drenthe/ Groningen/ Friesland/ Flevoland
5. How many years of professional experience do you have in orthotic care?
   a. Options: 0-1/ 1-3/3-5/ 5-10/10-20/ more than 20 years
6. Which diagnostic groups do you predominantly treat? (multiple answers possible)
   a. Options: Neuromuscular disease/ MS/ Stroke/Cerebral Palsy/ Trauma/ Spinal cord injury/ other (specify)
7. Prescribing leg orthoses is done at our clinic during a multidisciplinary consultation? (more professionals than physician-orthotist)
   a. Options: Always/Usually/Sometimes/ Never

**AFO users**

1. Year of birth
   a. open field
2. Gender
   a. option: Male / Female
3. In which province do you live?
   a. Options: Noord-Holland/ Zuid-Holland/ Utrecht/ Brabant/ Zeeland/ Limburg/ Gelderland/ Overijssel/ Drenthe/ Groningen/ Friesland/ Flevoland
4. What is your diagnosis?
   a. Options: Neuromuscular disease/ MS/ Stroke/Cerebral Palsy/ Trauma/ Spinal cord injury/ other (specify)
5. Can you stand on your toes on one leg?
   a. Yes, both sides
   b. Only on left leg
   c. Only on right leg
   d. No
6. For how many years do you use AFOs?
   a. Open field
7. Is your AFO custom-made?
   a. Options: Yes/No/ I don’t know
8. Can you describe your current AFO?
   a. Open field
