# Supplementary material for: A research priority setting for ankle-foot orthosis care in the Netherlands; a survey among rehabilitation specialists, orthotists, and orthotic users
Source: Prosthet Orthot Int. 2025 Oct 29;50(4):410–7. doi: 10.1097/PXR.0000000000000499 (PMC13440560; doi:10.1097/PXR.0000000000000499)
Supplement: Supplementary file 2 [file poi-50-410-s002.docx]

**Appendix 2. List of all research questions**

**Rehabilitation specialist**

| *When is an AFO indicated and which measurements are needed for indication setting?* | *Which orthosis is most suitable for the individual patient?* | *Effects of AFO use on muscle functioning and properties* | *Effects of AFO properties on gait* | *AFO Adherence; how often are AFOs used and which factors play a role in adherence.* | *Methods to evaluate AFO care* | *Effectiveness of AFOs on different aspects of functioning (impairment-activity-participation)* | *Efficiency of multidisciplinary AFO care and custom-made devices* |
| --- | --- | --- | --- | --- | --- | --- | --- |
| Which EVO is indicated and when? | Which orthosis works best for which patient (personalized AFO)? | How many hours should an AFO be worn to prevent calf muscle shortening? | What is effect of the weight of the orthosis on specific gait patterns? | What factors play a role in whether or not (sufficiently) schoolchildren wear an AFO? | What is the best way to evaluate the use of an orthosis? | To what extent do dynamic AFOs at night affect the improvement of mobility and thus sometimes daytime functioning? | Is there a difference in satisfaction/effect between an AFO fitted after an extensive analysis versus a pragmatically fitted AFO in the clinic? |
| Is there a minimum walking speed or step length required to optimally benefit from AFOs? | Which intervention contributes the most to improving walking efficiency? | What is the effect of foot correction within the AFO on foot development | What is effect of stiffness of the orthosis and the degree of mobility of the orthosis in the plantarflexion direction during walking? | Can the actual use of orthoses in daily life be predicted? | Are there quick evaluation options, without extensive gait analysis? | What is the effectiveness of AFOs regarding functioning (participation) | Does a trial AFO have added value in changing the concept? (A type of trial splint instead of a trial shoe) |
| What data is minimally needed to effectively set up an AFO prescription | What is the optimal splint for gait type 4 (Cerebral Palsy)? | What are the long-term effects on walking and muscle development | Which orthosis properties are the most important for orthosis provision? | What determines therapy adherence (parents/children)? | Is it possible to evaluate the orthosis remotely (video calling, questionnaires via an app)? | What is the effectiveness of AFOs regarding Socio-emotional functioning | What is the added value of a multidisciplinary gait clinic when prescribing AFOs for people with a neurological movement disorder? |
| How can we still use a dynamic splint for a (valgus) deformed foot? | What is the added value of a dynamic AFOs with, for example, Neuroswing hinges? | To what extent are night orthoses effective in preventing contractures? | Which AFOs (materials) are most durable? | How many of the prescribed splints (day/night) are actually worn by teenagers? | Is evaluation of orthosis in the recovery phase after a stroke required | What is the effect of AFOs on spasticity? | What are the costs of prefab versus custom-made AFOs when factoring in their lifespan. |
|  | When should one choose an AFO and when should one choose an orthopedic shoe with AFO functionality? | Which orthosis works best for preventing deformities during growth |  | What percentage of the AFOs are worn regularly vs. ending up in the closet? | When would you or would you not perform walking tests to determine the effect of an orthosis? |  |  |
|  | What is the best type of AFO for spasticity? |  |  | What causes the orthosis, despite careful selection and evaluation (in primary rehabilitation), to no longer be worn within one year? How often does this occur? | Which questionnaires can be used for orthotic evaluation? |  |  |
|  |  |  |  |  | How can we evaluate why something doesn't work, even if it is theoretically the best option. |  |  |

**Orthotist**

| *Which AFO is most suitable and how to determine the orthotic functions and properties of this AFO* | *Comparison of different AFO types on gait* | *Effects of AFO properties on gait* | *How to cast for the AFO and to correct the plaster model* | *Innovation in materials to reduce impact on environment, reduce health consequences and improve material duration.* | *Importance of AFO alignment on effectivity.* | *AFO Adherence; how often are AFOs used and which factors play a role in adherence.* | *Efficiency of guideline for custom-made AFOs and recycling of AFOs* |
| --- | --- | --- | --- | --- | --- | --- | --- |
| Is it possible to link functional assessment (MRC, weight, speed) to AFO design choice and stiffness? | How do different types of dynamic AFOS from various supplies differ in effect? | What is the effect of the AFO footplate and stiffness on walking? | Is it possible to take measure digitally for every case? | What are the possibilities to measure the rigidity of orthotic pieces produced with different 3D-printed materials? | What are the effects of improperly alignment of AFOs? | What factors influence whether someone uses an AFO or not, both in adults and in children where parents are involved? | What are the costs and benefits of expensive AFOs, such as those with costly hinges or prepreg materials? |
| How can you easily design and test the correct rigidity for an orthosis for a client? | Compare the functionality between dynamic and static AFOs regarding walking (i.e., with little or much resistance to dorsiflexion). | What is the effect of different materials on walking? | What are the advantages of taking digital measures? | Is it possible to develop a material with the same properties as carbon but less harmful to health. A type of synthetic fiber. | How critical is the shoe provision and precise execution of alignment for evaluating effectiveness? | What percentage of clients returns for a follow-up after the initial usage period if everything is going well? | How can used AFOs be repurposed or reused? |
| When should you choose a hinge in the ankle versus a carbon fiber orthosis with a return mechanism? |  | How energy-efficient are prepreg carbon ankle-foot orthoses? | Scanning versus manual methods: Which is better? | How to arrange carbon fibers to minimize the amount of carbon/thickness needed to achieve a rigid orthosis. |  | How satisfied and what are the experiences of AFO users? | What are the guidelines for using intelligent hinges in AFOs? |
| Which orthotic provision is effective for someone with spastic equinus varus foot (as seen in stroke patients), or what is the best approach to improve gait in these individuals? |  | How do different concepts/stiffness levels affect energy consumption. | Correction techniques and pressure points: What works and what doesn't? Is it possible to create a standardized procedure or checklist? | What are the most eco-friendly materials to use? |  | How do you assess adherence to therapy with an EVO? |  |

**AFO Users**

| *Provision of information during the AFO care process* | *Improving AFO functionality in daily life (example driving a car) and cosmetics* | *Possibilities to choose and test different AFOs before provision* | *Improvement of AFO comfort and fitting* | *Who is best suited to make the choice of concept and what is the influence of industry in this choice* | *Durability of AFO and AFO-components* | *Reducing the number of appointments and errors during the AFO fitting process* | *Improvement of AFO-shoe fitting and information on appropriate shoes* | *Scheduling of standard evaluation appointments* |
| --- | --- | --- | --- | --- | --- | --- | --- | --- |
| Could more information be provided to the users before the choice of the AFO is made? | How to avoid the negative affects of AFOs during daily life activities? | Would trying out AFOs before selecting one make a difference? | How can you better shape the mold/base for the orthoses so that less adjustment is needed after making the brace? | What are the reasons to choice for a certain AFO? | Could the AFO be made out of lighter materials? | Which appointments are necessary during the AFO care process? | What are the best shoes to wear with an AFO? | What would be the effect of standardly scheduling evaluation appointments? |
| Which characteristics should be discussed with the users? | Could an AFO with variable stiffness for different activities be developed? |  | How to make orthoses more comfortable during daily life? |  | Which materials are most durable for the straps and footplate? | Which techniques should be used to create the AFO to maximize fitting and comfort? | How do shoes affect the outcomes of the AFO? |  |
|  |  |  | Could an orthosis that is less visible and can be neatly hidden under clothing be developed? |  |  |  |  |  |
|  |  |  |  |  |  |  |  |  |
